# Variation in chilling tolerance for photosynthesis and leaf extension growth among genotypes related to the C_4_ grass *Miscanthus ×giganteus*

**DOI:** 10.1093/jxb/eru287

**Published:** 2014-07-19

**Authors:** Katarzyna Głowacka, Shivani Adhikari, Junhua Peng, Justin Gifford, John A. Juvik, Stephen P. Long, Erik J. Sacks

**Affiliations:** ^1^Institute for Genomic Biology, University of Illinois, 1206W. Gregory Dr., Urbana, IL 61801, USA; ^2^Institute of Plant Genetics, Polish Academy of Sciences, ul. Strzeszyńska 34, 60-479 Poznań, Poland; ^3^Department of Soil and Crop Science, Colorado State University Fort Collins, CO 80523-1170, USA

**Keywords:** Chilling, cold tolerance, electron transport, *Miscanthus ×giganteus*, *Miscanthus sacchariflorus*, *Miscanthus sinensis*, photosynthesis, plant breeding, *Saccharum officinarum*.

## Abstract

The goal of this study was to identify cold-tolerant genotypes within two species of *Miscanthus* related to the exceptionally chilling-tolerant C_4_ biomass crop accession: *M. ×giganteus* ‘Illinois’ (Mxg) as well as in other Mxg genotypes. The ratio of leaf elongation at 10 °C/5 °C to that at 25 °C/25 °C was used to identify initially the 13 most promising *Miscanthus* genotypes out of 51 studied. Net leaf CO_2_ uptake (*A*_sat_) and the maximum operating efficiency of photosystem II (Ф_PSII_) were measured in warm conditions (25 °C/20 °C), and then during and following a chilling treatment of 10 °C/5 °C for 11 d. Accessions of *M. sacchariflorus* (Msa) showed the smallest decline in leaf elongation on transfer to chilling conditions and did not differ significantly from Mxg, indicating greater chilling tolerance than diploid *M. sinensis* (Msi). Msa also showed the smallest reductions in *A*_sat_ and Ф_PSII_, and greater chilling-tolerant photosynthesis than Msi, and three other forms of Mxg, including new triploid accessions and a hexaploid Mxg ‘Illinois’. Tetraploid Msa ‘PF30153’ collected in Gifu Prefecture in Honshu, Japan did not differ significantly from Mxg ‘Illinois’ in leaf elongation and photosynthesis at low temperature, but was significantly superior to all other forms of Mxg tested. The results suggested that the exceptional chilling tolerance of Mxg ‘Illinois’ cannot be explained simply by the hybrid vigour of this intraspecific allotriploid. Selection of chilling-tolerant accessions from both of Mxg’s parental species, Msi and Msa, would be advisable for breeding new highly chilling-tolerant Mxg genotypes.

## Summary

Tetraploid *M. sacchariflorus* were identified as having equal chilling tolerance to *M.* ×*giganteus* ‘Illinois’, but *M. sinensis* were inferior. Heterosis did not explain the chilling tolerance of *M.* ×*giganteus*, but suboptimal ploidy reduced it.

## Introduction

During the last two decades, *Miscanthus* ×*gigantues* (Mxg) has become an important biomass crop for European and US bioenergy initiatives (Scurlock, 1999; Clifton-Brown *et al.*, 2004; Heaton *et al.*, 2008; Somerville *et al.*, 2010). By combining perenniality, rhizome function, and cold-tolerant C_4_ photosynthesis, Mxg is highly light, nitrogen, and water use efficient, which contributes to its exceptionally high biomass productivity even in cool temperate climates (Lewandowski *et al.*, 2000; Heaton *et al.*, 2008; Long and Spence, 2013). While many C_4_ plants are now known to survive cold conditions, Mxg appears exceptional in its ability to achieve the high productivity of C_4_ photosynthesis in cold climates. For example, in southeastern England, Mxg shoot productivity exceeded the highest values achieved by intensively managed C_3_ crops (Beale and Long, 1995). A comparative field study with a high-yielding maize cultivar (Illinois, USA) revealed that perenniality together with cold tolerance gave Mxg 59% more productivity than maize (Dohleman and Long, 2009). This was largely explained by the ability of Mxg to form photosynthetically competent leaves earlier in the year and maintain them later in the year. This allowed Mxg to capture 60% more solar energy than maize, and yet convert this into biomass with a similar efficiency, despite colder weather at the beginning and end of the growing season (Dohleman and Long, 2009).

A single genotype of Mxg collected from southern Honshu, Japan was initially introduced to Denmark in the 1930s and was then subsequently distributed throughout Europe and North America (Greef and Deuter, 1993; Hodkinson *et al.*, 2002; Głowacka *et al.*, 2014). This cultivar is a triploid sterile hybrid between diploid *M. sinensis* (Msi) and tetraploid *M. sacchariflorus* (Msa) (Greef and Deuter, 1993; Linde-Laursen, 1993; Hodkinson *et al.*, 2002). Hodkinson and Renvoize (2001) defined the nothospecies, *Miscanthus* ×*gigantues* Greef & Deu. ex Hodkinson & Renvoize, as a hybrid between Msi and Msa, a designation that could include an infinite number of genotypes. However, almost all trials and production fields of Mxg appear to be from the single clone first introduced to Denmark, though limited information about new natural Mxg accessions and those produced by controlled crosses is beginning to appear (Nishiwaki *et al.*, 2011; Jeżowski *et al.*, 2011; Dwiyanti *et al.*, 2013). Additionally, new genotypes of Mxg have recently been bred at the Univeristy of Illinois via controlled crosses (Chae *et al.*, 2014). The first replicated trials of Mxg in the USA were undertaken in Illinois by Heaton *et al.* (2008), and this has since become known as the ‘Illinois’ clone. While the ‘Illinois’ clone has been shown to achieve high productivity and is being used in small- and medium-scale production operations, it does have limitations. First, a single clone is highly vulnerable to potential diseases and pests. Secondly, its origin in southern Honshu is far south of the northern limit of *Miscanthus* in eastern Asia. Therefore, it is likely that there is greater cold tolerance in the germplasm that would allow the development of cultivars for colder climates than that of England and Illinois or that would have a longer potential growing season within these climates. Thus, there is a strong need for breeding efforts to produce and evaluate new Mxg genotypes. Indeed since the ‘Illinois’ clone is a sterile triploid hybrid, finding potential parent lines with at least an equivalent chilling tolerance will be important for developing new hybrids adapted to temperate environments. Such genotypes would be useful not only for *Miscanthus* breeding but also for improving cold tolerance in sugarcane. *Miscanthus* has been hybridized with and subsequently backcrossed to sugarcane, facilitating introgression of disease tolerance genes (Chen *et al.*, 1993). It would therefore seem likely that the same approach could be used to introgress cold tolerance from selected *Miscanthus* genotypes into sugarcane.

To date, most studies of chilling-tolerant photosynthesis in *Miscanthus* have focused on the single commercial genotype of Mxg (Beale *et al.*, 1996; Naidu *et al.*, 2003; Naidu and Long, 2004; Farage *et al.*, 2006; Wang *et al*., 2008a, b). An exception was an analysis of the response of photosynthesis to chilling (12 °C for 12h) for two Msi hybrids, one tetraploid Msa and one Mxg, by Purdy *et al.* (2013). Additionally, Clifton-Brown and Jones (1997) found that genotypic variation in leaf expansion at 5 °C relative to 20 °C shows a strong correspondence to final yield in *Miscanthus*.

Exposure to chilling temperatures (>0≤14 °C) has reversible as well as irreversible effects on C_4_ photosynthesis. Chilling lowers the capacity for CO_2_ assimilation and, in the absence of adequate alternative pathways for dissipating absorbed excitation energy, can lead to reversible photoinhibition or irreversible photooxidation (Long, 1983; Long *et al.*, 1994; Allen and Ort, 2001). Mxg has been shown to avoid this damage by increasing photosynthetic capacity under chilling conditions (Wang *et al.*, 2008b) and by increasing its capacity for non-photochemical quenching of absorbed light energy (Farage *et al.*, 2006).

In Mxg this chilling tolerance might come from one or both parents, or be a result of hybrid vigour. It might also be expected to come from polyploidy, since this could amplify genes conferring chilling tolerance. The recent development of a hexaploid by chromosome doubling of the ‘Illinois’ clone, as well as the discovery of new triploid and diploid forms of Mxg, provide an opportunity to test these hypotheses. When considering variation in overall chilling tolerance among genotypes, variation for chilling tolerance of leaf extension growth must also be considered, since both leaf extension and photosynthesis must have similar chilling tolerance to provide a productivity advantage to a cultivar. In *Miscanthus*, leaf extension in low temperature is indicative of chilling tolerance (Clifton-Brown and Jones, 1997), and specific leaf area can correlate with CO_2_ assimilation rate at low temperature (U. Jørgensen, personal communication).

The foregoing gives rise to a series of questions about genetic variation in relatives of Mxg that are investigated here. (i) What variation in chilling tolerance can be found in different genotypes of Mxg and in Mxg’s parental species? (ii) Can chilling tolerance equal to or better than that of the Mxg ‘Illinois’ be found? (iii) From which of the parental Mxg species is chilling tolerance most probably inherited? (iv) Is there an association between increasing ploidy level and chilling tolerance? To address these questions, low temperature leaf elongation of Mxg ‘Illinois’ together with 50 additional *Miscanthus* genotypes belonging to seven species-ploidy groups were determined, and a subset were further evaluated for photosynthetic capacity during and after chilling conditions.

## Materials and methods

### Plant material

A total of 51 *Miscanthus* accessions were studied (Table 1): three diploid Msa, 10 tetraploid Msa, 27 diploid Msi, five tripolid Mxg, three colchicine-induced polyploids (5*x*–6*x*) of Mxg ‘Illinois’ (Chae *et al.*, 2013), one diploid Mxg, one *M. oligostachyus* ‘Purpurascens’, and a *Miscanthus* accession of unknown provenance named *Miscanthus* sp. ‘PF1-7’. Most of the Msi genotypes were cultivars commercially available in the USA, and chosen based on spring and/or autumn hardiness, and other features related to cold tolerance, namely early emergence, late flowering, and remaining green after autumn frost (C. Kaiser and E. Sacks, unpublished data). Most of the tetraploid Msa studied originated from Gifu Prefecture, Honshu, Japan. Among the triploid Mxg accessions were the ‘Illinois’ clone, and four new triploid Mxg genotypes obtained from a cross [Msa (4*x*) ‘Bluemel Giganteus’×Msi (2*x*) var. *condensatus* ‘Cabaret’] made at the University of Illinois (Chae *et al.*, 2014). Two sugarcane (*Saccharum officinarum*) cultivars and two *Zea mays* lines were included as controls.

**Table 1.** Miscanthus, *sugarcane and maize accessions included in the leaf elongation study*

Name	Accession identifier	Source
***M. sacchariflorus* (2*x*)**		
‘PMS-075’	PMS-075	J. Peng, Wuhan Botanical Garden, China
‘Robustus-Bluemel’	UI10-00009	Kurt Bluemel, INC nursery, MD, USA
var. *lutarioriparius* ‘PF30022’	UI11-00031	New Energy Farms, Canada
***M. sacchariflorus* (4*x*)**		
‘Bluemel Giganteus’	UI10-00117	Kurt Bluemel, INC nursery, MD, USA
‘EMI-5’	MATEREC11	U. Jørgensen, Aarhus Univ., Denmark← M. Deuter, Tinplant, Germany
‘Gotemba Gold’	UI11-00005	Glasshouse Works nursery, OH, USA
‘PF30150’	UI11-00032	Honshu, Japan by New Energy Farms, Canada
‘PF30151’	UI11-00033	Honshu, Japan by New Energy Farms, Canada
‘PF30153’	UI11-00035	Honshu, Japan by New Energy Farms, Canada
‘PF30154’	UI11-00036	Honshu, Japan by New Energy Farms, Canada
‘PF30155’	UI11-00037	Honshu, Japan by New Energy Farms, Canada
‘PF30156’	UI11-00038	Honshu, Japan by New Energy Farms, Canada
‘PF30157’	UI11-00039	Honshu, Japan by New Energy Farms, Canada
***M. sinensis* (2*x*)**		
‘Autumn Light’	UI10-00025	Emerald Coast Growers nursery, FL, USA
‘Blondo’	UI11-00017	Kurt Bluemel, INC nursery, MD, USA
‘Burgander’	UI10-00035	Walla Walla Nursery Co., WA, USA
‘Dixieland’	UI10-00036	Emerald Coast Growers nursery, FL, USA
‘Emerald Shadow’	UI10-00038	Kurt Bluemel, INC nursery, MD, USA
‘Emmanuel LePage’	UI10-00039	Kurt Bluemel, INC nursery, MD, USA
‘Ferner Osten’	UI10-00040	Kurt Bluemel, INC nursery, MD, USA
‘Gracillimus’	UI10-00048	Emerald Coast Growers nursery, FL, USA
‘Huron Sentinel’	UI10-00057	Paradise Garden nursery, TN, USA
‘Huron Sunrise’	UI10-00058	Paradise Garden nursery, TN, USA
‘Kleine Silberspinne’	UI12-00008	Earthly Pursuits nursery, MD, USA
‘Morning Light’	UI10-00071	Walla Walla Nursery Co, WA, USA
‘Nippon’	UI10-00074	Emerald Coast Growers nursery, FL, USA
‘November Sunset’	UI10-00075	Kurt Bluemel, INC nursery, MD, USA
‘Roland’	UI10-00080	Earthly Pursuits nursery, MD, USA
‘Sarabande’	UI11-00010	Emerald Coast Growers nursery, FL, USA
‘Silberfeder’	UI12-00006	Earthly Pursuits nursery, MD, USA
‘Teshio’	UI11-00003	T. Yamada, Hokkaido Univ., Japan
‘Tripple Brook Farm’	UI10-00011	Tripple Brook Farm nursery, MA, USA
‘Undine’	UI12-00009	Earthly Pursuits nursery, MD, USA
‘Uryu’	UI11-00004	T. Yamada, Hokkaido Univ., Japan
var. *condensatus* ‘Cabaret’	UI10-00012	Emerald Coast Growers nursery, FL, USA
var. *condensatus* ‘Cosmopolitan’	UI10-00015	Emerald Coast Growers nursery, FL, USA
var. *purpurascens*	UI10-00019	Kurt Bluemel, INC nursery, MD, USA
var*. transmorrisonensis*	UI10-00106	Walla Walla Nursery Co, WA, USA
‘Variegatus’	UI10-00097	Emerald Coast Growers nursery, FL, USA
‘Zebrinus’	UI11-00016	Kurt Bluemel, INC nursery, MD, USA
***M.* ×*giganteus* (3*x*)**		
‘Illinois’	UI10-00107	T. Voigt, UI, USA← Chicago Botanic Garden, USA
‘10UI-032.001’	10UI-032.001	J. Gifford, J Juvik & E. Sacks, UI, USA
‘10UI-032.002’	10UI-032.002	J. Gifford, J Juvik & E. Sacks, UI, USA
‘10UI-032.003’	10UI-032.003	J. Gifford, J Juvik & E. Sacks, UI, USA
‘10UI-032.004’	10UI-032.004	J. Gifford, J Juvik & E. Sacks, UI, USA
***M.* ×*giganteus* colchicine-induced polyploids**		
‘Illinois-5*x*.02 (Mxg2*x*-2)’	UI10-00110	W. Chae & J. Juvik, UI, USA
‘Illinois-6*x*.01 (Mxg2*x*-1)’	UI10-00109	W. Chae & J. Juvik, UI, USA
‘Illinois-6*x*.07 (Mxg2*x*-7)’	UI10-00113	W. Chae & J. Juvik, UI, USA
***M.* ×*giganteus* (2*x*)**		
var. *purpurascens* ‘Herkules’	UI10-00018	Kurt Bluemel, INC nursery, MD, USA
**Other *Miscanthus* accessions**		
*Miscanthus* sp. ‘PF1-7’	UI11-00046	New Energy Farms, Canada
***M. oligostachyus* ‘Purpurascens’**	UI10-00005	Walla Walla Nursery Co, WA, USA
Control sugarcane and maize lines		
*S. officinarum* ‘Louisiana Purple’	PI495639	USDA-NPGS, USA
*Saccharum* sp. ‘L79-1002’	PI651501	USDA-NPGS, USA
*Z. mays* hybrid ‘DK065-44VT3’	DK065	Dekalb Brand Corn, USA
*Z. mays* inbred line ‘FR1064’	FR1064	S. Moose, UI, USA← Illinois Foundation Seeds, IL, USA

Clonal divisions of *Miscanthus* and sugarcane accessions as well as *Z. mays* seedlings were grown in square pots of 1 litre volume (mini-treepot # MT38; Stuewe & Sons, Tangent, OR, USA) containing a peat-, bark-, and perlite-based growing medium (Metro-Mix 900; Sun Gro Horticulture, Agawam, MA, USA). After planting or sowing, an all-purpose slow release fertilizer was added following the manufacturer’s instructions (Osmocote Classic, 8–9 mo 13-13-13; Everris NA, Inc., Dublin, OH, USA), and additional iron added (ferrous sulphate heptahydrate; QC Corporation, Girardeau, MO, USA). Prior to transfer to controlled-environment cabinets, plants were grown in a controlled environment greenhouse at ~25 °C. Throughout, soil moisture content was maintained by watering to field capacity daily.

### Leaf elongation

All 55 accessions (51 *Miscanthus*, two sugarcane, and two maize) were evaluated for leaf elongation under chilling temperatures, enabling those with the greatest and least chilling tolerance to be identified efficiently for further study. Plants were transferred to controlled-environment chambers (BioChambers GRC-40; Conviron, Winnipeg, Manitoba, Canada) with a 12h day/12h night cycle under 1000 μmol photons m^–2^ s^–1^, a constant temperature of 25 °C, and relative humidity of 50%. After 10 d acclimation to the controlled-environment chamber, three out of six plants from each accession were selected at random, and transferred to a second identical controlled-environment chamber, but with a 10 °C/5 °C day/night temperature. All other environmental conditions were unchanged. To avoid confounding genotype with any undetected variation, the position of each plant within the chamber was changed following a randomized design every second day. Data were collected on the uppermost leaf on the stem in which the ligule had not emerged from the sheath of the preceding leaf (i.e. growing leaves). The distance from the top edge of the pot to the tip of the chosen leaf was measured using a ruler. For each plant, leaf growth was calculated by subtracting the measurement on day 0 of the treatment from the measurements collected on subsequent days. In the 10 °C/5 °C treatment, developing leaves were measured every second day, and every day in the control.

### Gas exchange and chlorophyll fluorescence

**Fig. 1.**
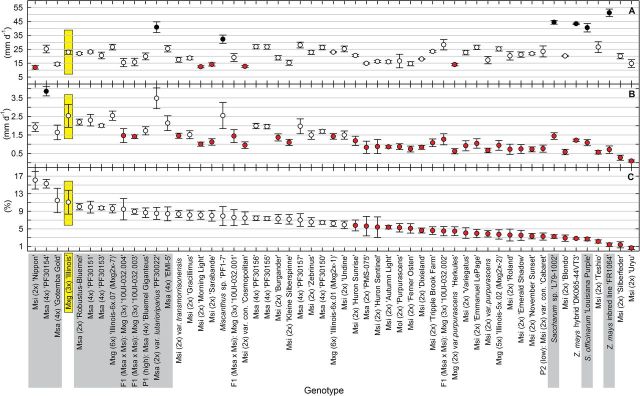
(A) Rate of leaf elongation at warm temperature and (B) chilling temperature, and (C) percentage of leaf elongation retained in chilling temperature relative to warm temperature for 51 *Miscanthus* accessions and four control sugarcane and maize lines. In chilling, developing leaves were measured during 14 d every other day, while for warm conditions data were collected during 7 d every day. The growing conditions were 10 °C/5 °C (chilling) or 25 °C/25 °C (warm) day/night and a 12h day/12h night cycle under 1000 μmol photons m^–2^ s^–1^. In all panels, accessions are ordered according to percentage of leaf elongation retained in chilling temperature relative to warm temperature (from the highest to the lowest, C). For each treatment stage, open symbols indicate no significant differences and filled symbols indicate significantly faster (black) or slower (red) elongation in comparison with *M.* ×*giganteus* (Mxg) (3*x*) ‘Illinois’ (yellow frame) based on Dunnett’s test (*P*≤0.1). Rates of leaf elongation for Mxg (3*x*) ‘Illinois’ were: (A) 22.87 (mm d^–1^); (B) 2.53 (mm d^–1^); (C) 11.08 (%). Data are mean ±SE (*n*=3). Grey- and yellow-highlighted genotypes were selected for a subsequent experiment to study chilling-tolerant photosynthesis. F1, the first generation of Msa×Msi hybrids; Mol*, M. oligostachyus*; Msa, *M. sacchariflorus*; Msi, *M. sinensis*; Mxg, *M.* ×*giganteus*; con., *condensatus*; P1 (high), parent 1 of interspecific Msa×Msi hybrids (Msa with high chilling tolerance); P2 (low), parent 2 of interspecific Msa×Msi hybrids (Msi with low chilling tolerance).

The high-throughput leaf elongation experiment served as a preliminary screen to select the most cold-tolerant genotypes for a more detailed but lower throughput assessment of photosynthetic response to chilling. A subset of 13 accessions, chosen from those which showed the greatest percentage of leaf elongation retained in chilling temperature relative to warm temperature and three negative controls (16 accessions in total; Fig. 1, grey- and yellow-marked accessions), was analysed for photosynthetic capacity under chilling conditions. Four plants of each accession were gown at 25 °C/20 °C (warm) in each of two controlled-environment chambers (Conviron PGR15; Controlled Environments) for 10 d, after which the temperature in both chambers was lowered to 10 °C/5 °C (chilling) day/night for 11 d, and then returned to 25 °C/20 °C. This period was chosen to mimic the type of chilling that might develop during spring after leaf emergence or expanded leaves in the autumn. Loss of efficiency during these periods greatly limits realization of the superior efficiency of C_4_ relative to C_3_ photosynthesis in temperate climates (Long and Spence, 2013). The position of plants within the chambers was changed, as described for the leaf elongation study. In both chambers a 14h day/10h night cycle with 1000 μmol photons m^–2^ s^–1^ and relative humidity of 65% was maintained. Leaf photosynthetic gas exchange and modulated chlorophyll fluorescence were measured *in situ* on the most recent fully expanded attached leaves, as judged by ligule emergence, with an open gas exchange system incorporating differential infrared CO_2_ and water vapour analysers (LI-6400; LI-COR, Lincoln, NE, USA). In this system, the leaf was enclosed in a controlled-environment cuvette which tracked the light, temperature, and humidity in the controlled-environment chamber. Chlorophyll pulse amplitude modulated fluorescence was measured simultaneously with a fluorometer incorporated into the cuvette lid (LI-6400–40; LI-COR, Inc.). Measurements were conducted under ambient air (21% O_2_) at 390 μmol mol^–1^ CO_2_ concentration, 1000 μmol m^–2^ s^–1^ photon flux, and 65% relative humidity. Leaf temperature was maintained at the growth temperature for each accession and treatment. Actinic light was supplied by light-emitting diodes (90% red light, 630nm; 10% blue light, 470nm). To maximize the fluorescence emissions, the fluorometer parameters (e.g. flash intensity and duration) were adjusted and the multiphase protocol was used. These measurements were made in warm conditions (25 °C) just prior to the chilling treatment, immediately after the temperature was reduced to 10 °C (day 0), during each of the 11 d at 10 °C (except days 6, 8, and 10 when the measurements were not taken), and finally 1 d after transferring the plants back to 25 °C (12th day of the experiment—recovery). All measurements were taken during the day on light-adapted leaves until steady state (20–50min). On day 0, in order to obtain measurements for each plant immediately after reducing the temperature to 10 °C, each pot was individually transferred from the 25 °C growing chamber to a growing chamber at 10 °C; after the leaves reached steady-state readings, gas exchange parameter were recorded. For each accession, four replicate plants were measured for each treatment. From these measurements, leaf net CO_2_ uptake per unit leaf area (*A*), stomatal conductance to water vapour (*g*_s_), intercellular CO_2_ concentration (*c*_i_), the quantum yield of photosystem II (Ф_PSII_), and rate of whole chain electron transport (*J*) were calculated, as described previously (Bernacchi *et al.*, 2003).

### Data analysis

All statistical analyses were performed with SAS v. 9.3 (SAS Institute, Cary, NC, USA). Leaf elongation was regressed against time to determine the best-fitting equation to describe the rate of leaf growth by using linear regression analysis in PROC REG. The significance of genotype and treatment on all measures (leaf elongation rate, *A*, *g*_s_, *c*_i_/*c*_a_, and Ф_PSII_) was determined by two-way analysis of variance (ANOVA) in PROC GLM; genotype and treatment were fixed effects. Where a significant effect of genotype or species-ploidy group was detected, Dunnett’s test was used to determine which genotypes differed significantly from the Mxg ‘Illinois’. If significant interactions between genotype and treatment were observed, then treatment effects within each genotype were separately evaluated for significance by Tukey’s test.

## Results

### Genotypic variation in leaf elongation

Mxg (3*x*) ‘Illinois’, the reference chilling-tolerant accession, showed a 9-fold decrease in leaf elongation rate from 22.9mm d^–1^ at 25 ºC to 2.5mm d^–1^ at 10 °C/5 °C (Fig. 1A, B). Of the 50 *Miscanthus* genotypes analysed, two groups were identified, one with 17 accessions that were not significantly different from Mxg (3*x*) ‘Illinois’ and the other with 32 accessions that showed a significantly greater reduction in leaf extension growth (*P*≤0.1; Fig. 1B). Notably, one accession, Msa ‘PF30154’, significantly exceeded the leaf elongation of Mxg (3*x*) ‘Illinois’ by 1.5-fold when grown at chilling temperature (Fig. 1B). There was considerable variation between *Miscanthus* accessions, with Msa ‘PF30154’ showing 43-fold greater leaf extension growth at 10 °C/5 °C than the worst performing accession, diploid Msi ‘Uryu’ (Fig. 1B). The two cultivars of *Z. mays* and two accessions of *S. officinarum* showed some of the lowest extension rates at 10 °C/5 °C, but comparable with the worst performing *Miscanthus* accessions. However, at 25 °C, all four maize and sugarcane controls had significantly greater leaf elongation rates than Mxg (3*x*) ‘Illinois’. Only two *Miscanthus* accessions matched these rates, Msa var. *lutarioriparius* ‘PF30022’ and *Miscanthus* sp. ‘PF1-7’. However, Msa var. *lutarioriparius* showed only a 12-fold decline in leaf elongation in the chilling treatment, compared with >30-fold declines for the *Saccharum* accessions and the *Z. mays* cultivars.

**Fig. 2.**
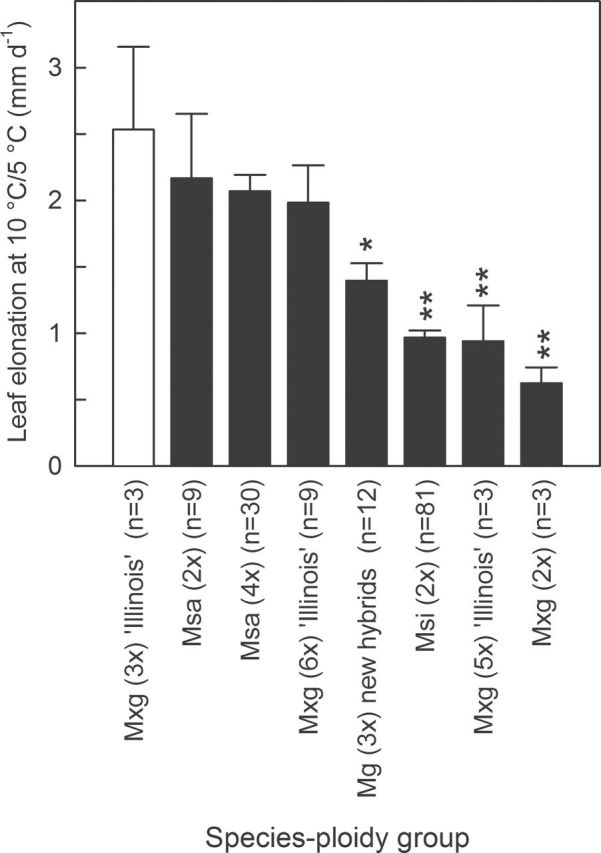
Influence of species-ploidy group on leaf elongation at 10 °C/5 °C. Leaves were measured during 14 d every other day for plants growing at 10 °C/5 °C day/night temperature and a 12h day/12h night cycle under 1000 μmol photons m^–2^ s^–1^. Species-ploidy groups are ordered from highest to lowest leaf elongation at 10 °C/5 °C. Asterisks indicate significantly lower leaf elongation in comparison with *M.* ×*giganteus* (3*x*) ‘Illinois’ (2.53mm d^–1^) based on Dunnett’s test (^†^ *P*≤0.1; *P≤0.05; **P≤0.01). Data are the mean +SE (*n* varied from 3 to 81 depending on group). Msa, *M. sacchariflorus*; Msi, *M. sinensis*; Mxg, *M.* ×*giganteus*.

At 10 °C/5 °C, all 10 tested tetraploid Msa and two hexaploid Mxg were not significantly different from Mxg (3*x*) ‘Illinois’ (Fig. 1C). Two of the three diploid Msa were also not significantly different from Mxg (3*x*) ‘Illinois’. In contrast to the Msa accessions, approximately two-thirds of the Msi accessions had significantly lower rates of leaf elongation at chilling temperatures than Mxg (3*x*) ‘Illinois’ (Figs 1, 2; Supplementary Fig. S1 available at *JXB* online).

Among four new triploid Mxg obtained from the cross, Msa (4*x*) ‘Bluemel Giganteus’×Msi (2*x*) var. *condensatus* ‘Cabaret’, three of the progeny and their Msa parent were not significantly different from Mxg (3*x*) ‘Illinois’ for percentage of leaf elongation retained in chilling temperature relative to warm temperature (Fig. 1C). However, the Msi parent (P2 low; Fig. 1), was among the least cold-tolerant accessions studied (similar to sugarcane and maize) as assessed by leaf elongation.

### Genotypic variation in photosynthetic gas exchange and chlorophyll fluorescence

**Fig. 3.**
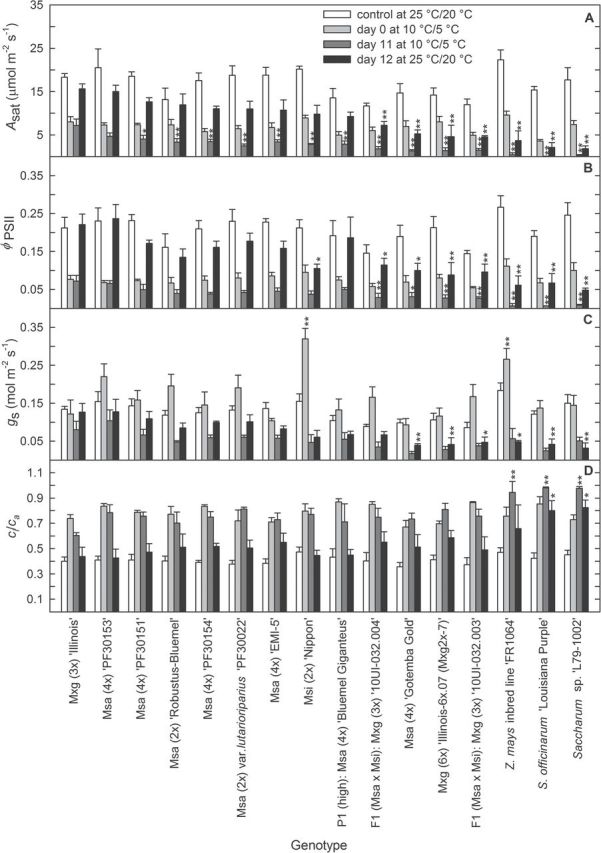
(A) Leaf CO_2_ uptake rate (*A*_sat_), (B) quantum yield of photosystem II (Ф_PSII_), (C) stomatal conductance to water vapour (*g*_s_), and (D) ratio of intercellular to atmospheric CO_2_ concentration (*c*_i_/*c*_a_) for warm conditions prior to chilling treatment, after transfer of plants to chilling (day 0), on the 11th day of chilling treatment and 1 d after transfer of plants back to warm conditions (12th day of the experiment—recovery). Plants were grown at 10 °C/5 °C (chilling) or 25 °C/20 °C (warm) day/night, with a 14h day/10h night cycle under 1000 μmol photons m^–2^ s^–1^. Measurements were taken during the daytime. In all panels, accessions are ordered according to *A*_sat_ on day 12 of the experiment [from the highest to lowest; A, fourth bar (black fill) for each genotype]. For each treatment stage, asterisks indicate significant differences in comparison with *M.* ×*giganteus* (3*x*) ‘Illinois’ based on Dunnett’s test (*P≤0.05; **P≤0.01). Subsequent time point values for Mxg (3*x*) ‘Illinois’ were: (A) 18.29, 7.99, 7.19, and 15.63 (μmol m^–2^ s^–1^); (B) 0.21, 0.08, 0.07, and 0.22 (dimensionless); (C) 0.13, 0.12, 0.08, and 0.13 (mol m^–2^ s^–1^); (D) 0.40, 0.74, 0.60, and 0.44 (dimensionless). Data are the mean ±SE (*n*=4). F1, the first generation of Msa×Msi hybrids; Msa, *M. sacchariflorus*; Msi, *M. sinensis*; Mxg, *M.* ×*giganteus*; P1 (high), parent 1 of interspecific Msa×Msi hybrids (Msa with high chilling tolerance).

Tetrapliod Msa ‘PF30153’ did not differ significantly from the cold-tolerant control, Mxg (3*x*) ‘Illinois’, for *A*_sat_ and Ф_PSII_ in both warm and cool conditions (Fig. 3). For Mxg (3*x*) ‘Illinois’ grown at 25 ºC/20 ºC, *A*_sat_ was 18.3 μmol m^–2^ s^–1^ and Ф_PSII_ 0.212, while for Msa ‘PF30153’ the respective values were slightly higher at 20.52 μmol m^–2^ s^–1^ and 0.231. After 11 d at 10 ºC/5 ºC, *A*_sat_ was 7.19 μmol m^–2^ s^–1^ and Ф_PSII_ 0.072 for Mxg (3*x*) ‘Illinois’, whereas for Msa ‘PF30153’ the respective values were 4.72 μmol m^–2^ s^–1^ and 0.066, which were not significantly different from those of Mxg (3*x*) ‘Illinois’. In contrast, the accessions of sugarcane and *Z. mays* showed a 94–99.5% reduction in *A*_sat_ and 88–91% reduction in Ф_PSII_ relative to Mxg (3*x*) ‘Illinois’ after 11 d at 10 ºC/5 ºC.

**Fig. 4.**
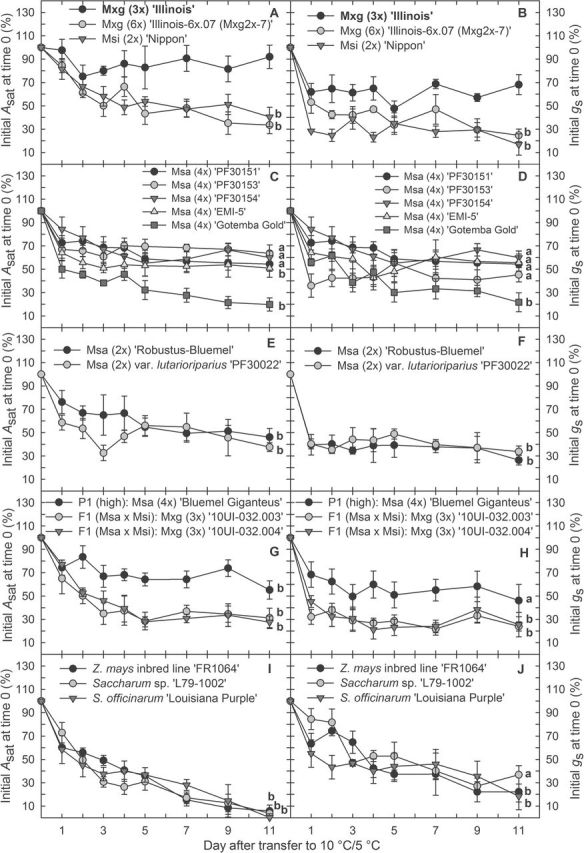
Changes in (A, C, E, G, I) leaf CO_2_ uptake rate (*A*_sat_) and (B, D, F, H, J) stomatal conductance to water vapour (*g*_s_) following transfer of plants from warm to chilling conditions. Values are expressed as a percentage of initial rates at time 0. (A and B) Accessions at different ploidy levels; (C and D) tetraploid *M. sacchariflorus* (Msa); (E and F) diploid Msa; (G and H) interspecific hybrids (F1) and their Msa parent (P1; high); (I and J) negative controls. Plants were grown at 25 °C/20 °C (warm) or 10 °C/5 °C (chilling) day/night, and a 14h day/10h night cycle under 1000 μmol photons m^–2^ s^–1^. Measurements were taken during the daytime. Data are the mean ±SE (*n*=4). Lower case letters indicate: ‘a’, non-significant differences; or ‘b’, significant differences in comparison with *M.* ×*giganteus* (3*x*) ‘Illinois’ (bold) on the 11th day after transfer to 10 °C/5 °C based on Dunnett’s test (*P*≤0.05). Values for Mxg (3*x*) ‘Illinois’ on the 11th day of chilling treatment were: (A) 92.02%; (B) 68.18%. F1, the first generation of Msa×Msi hybrids; Msa, *M. sacchariflorus*; Msi, *M. sinensis*; Mxg, *M.* ×*giganteus*; P1 (high), parent 1 of interspecific Msa×Msi hybrids (Msa with high chilling tolerance).

**Fig. 5.**
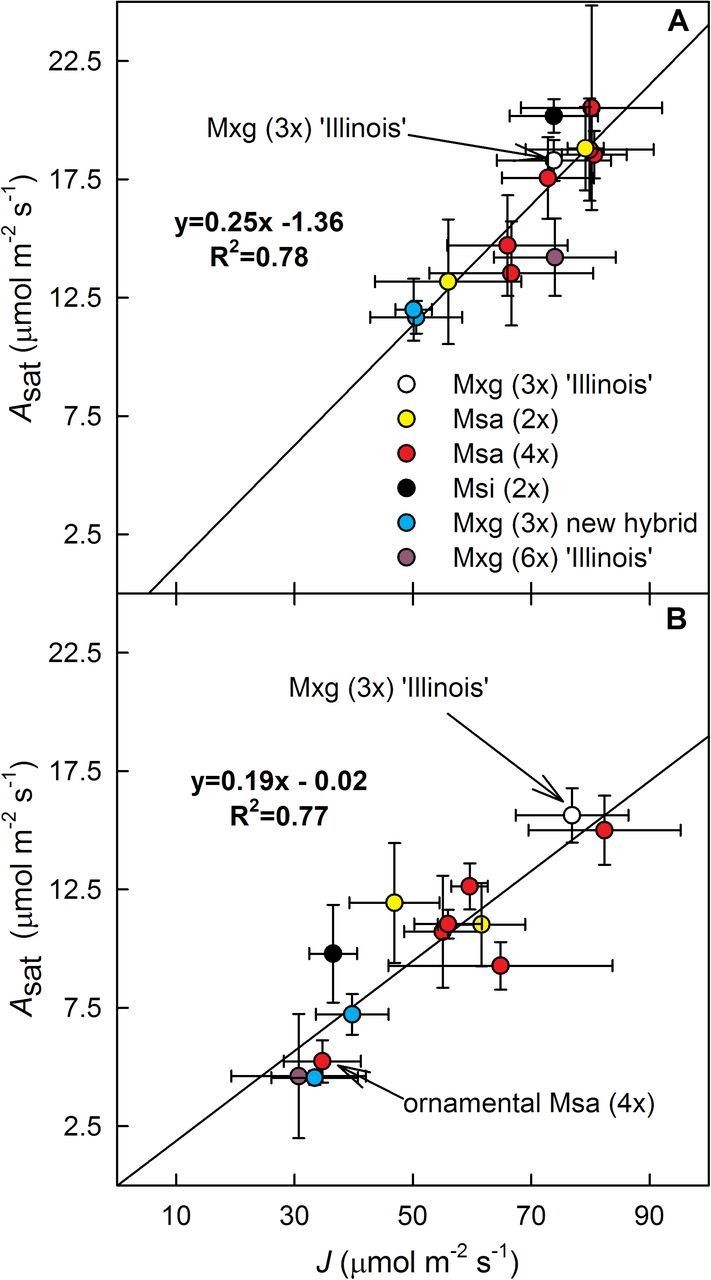
Electron transport rates (*J*) in relation to CO_2_ uptake rates (*A*_sat_) for: (A) plants grown in control warm conditions prior to chilling treatment and (B) for the recovery day when plants after 11 d chilling treatment were transferred back to warm conditions. Plants were grown at 10 °C/5 °C (chilling) or 25 °C/20 °C (warm) day/night, and a 14h day/10h night cycle under 1000 μmol photons m^–2^ s^–1^. Measurements were taken during the day. Data are mean ±SE (*n*=4). Msa, *M. sacchariflorus*; Msi, *M. sinensis*; Mxg, *M.* ×*giganteus*.

Three main patterns of loss of photosynthetic capacity could be observed across the germplasm studied, over the 11 d following transfer to 10 ºC/5 ºC. These may be characterized as nearly full acclimatization, partial acclimatization, and failure to acclimatize (Fig. 4; Supplementary Fig. S3 at *JXB* online). For Mxg (3*x*) ‘Illinois’, *A*_sat_ and Ф_PSII_ declined for the first 2–3 d at 10 ºC/5 ºC, followed by a recovery over the following days to stabilize at a level of 91–92% of the rates on initial transfer to 10 °C/5 ºC (Fig. 4A, C). Msa (4*x*) ‘PF30153’ was the only clone studied which showed a similar pattern of response and was not significantly different from Mxg (3*x*) ‘Illinois’ with respect to *A*_sat_ at the end of the chilling treatment. Both Mxg (3*x*) ‘Illinois’ and Msa ‘PF30153’ recovered fully 1 d after transfer back to 25 °C, showing ~87% and 84%, respectively, of the pre-chilling *A*_sat_ (Supplementary Fig. S3A). Additionally, two other tetraploid Msa, ‘PF30151’ and ‘PF30154’, did not differ significantly from Mxg (3*x*) ‘Illinois’ with respect to relative *A*_sat_ at the end of the chilling treatment (*A*_sat_ calculated as a percentage of initial values at day 0; Fig. 4). Compared with Mxg (3*x*) ‘Illinois’, all other *Miscanthus* clones showed a significantly lower *A*_sat_ over the 11 d of chilling (Figs 3, 4). Although seven other genotypes, six Msa and Msi ‘Nipon’, showed a significantly lower *A*_sat_ over the 11 d at 10 °C/5 °C compared with Mxg, on return to warm conditions they recovered an *A*_sat_ after 1 d that was not significantly lower than the value for Mxg (3*x*) ‘Illinois’ (Fig. 3A). All other lines showed a significantly greater loss of photosynthetic capacity during the 11 d at 10 °C/5 °C and on return to warm conditions, although none showed as large a loss as the *Z. mays* and two *Saccharum* spp. lines (Figs 3A, 4; Supplementary Fig. S2A). Several lines showed an increase in stomatal conductance on initial transfer to 10 °C/5 °C (Fig. 3; Supplementary Fig. S2). All lines showed an increase in *c*_i_/*c*_a_ on transfer to 10 °C/5 °C, but only *Z. mays* and the two *Saccharum* spp. lines showed a significant increase in *c*_i_/*c*_a_ over the 11 d of chilling compared with Mxg (3*x*) ‘Illinois’. Both *Saccharum* spp. lines also showed an increase in *c*_i_/*c*_a_ on return to 25 °C/20 °C relative to pre-chilling levels. In general, the other Mxg genotypes tested, including the hexaploid derived from the Mxg (3*x*) ‘Illinois’, were among the less chilling-tolerant lines, with respect to gas exchange measures (Figs 3, 4; Supplementary Fig. S2). There was a close correlation between *A*_sat_ and whole chain electron transport (*J*) calculated from Ф_PSII_ both before and after the chilling treatment (Fig. 5). However, the *A*_sat_/*J* declined ~34% across the genotypes as a result of the chilling treatment (Fig. 5).

## Discussion

### What variation in chilling tolerance can be found within Mxg?

Mxg (3*x*) ‘Illinois’, which is the same clone that has been widely trialled in Europe (Hodkinson *et al.*, 2002; Głowacka *et al.*, 2014), has exceptional chilling tolerance of photosynthesis and leaf elongation, underlying its high productivity in cool climates (Beale and Long, 1995; Beale *et al.*, 1996; Wang *et al.*, 2008; Dohleman and Long, 2009; Purdy *et al.*, 2013; Arundale *et al.*, 2014). However, the basis of this chilling tolerance remains unclear. One possibility is that the performance of this clone at chilling temperatures results from hybrid vigour rather than simple inheritance from its Msa and Msi parents. To test this hypothesis, new triploid hybrids were generated by crossing Msa (4*x*) ‘Bluemel Giganteus’ and Msi (2*x*) var. *condensatus* ‘Cabaret’. It was known from previous experience that the ‘Cabaret’ parent was insufficiently hardy to over-winter undamaged in cold-temperate environments, such as in central Illinois. Four highly vigorous full-sibling triploid progeny were obtained, all of which had significantly lower leaf extension rates at low temperature relative to Mxg (3*x*) ‘Illinois’, and the two best siblings for leaf elongation also had significantly lower leaf CO_2_ uptake rates and whole-chain electron transport when grown at 10 °C/5 °C compared with Mxg (3*x*) ‘Illinois’. Thus, hybrid vigour appears to have been insufficient to account for chilling tolerance in Mxg.

Doubling the chromosome number in triploid Mxg ‘Illinois’ resulted in hexaploid lines with significantly lower chilling tolerance with respect to *A*_sat_ and Φ_PSII_ (Figs 3–5; Supplementary Fig. S3 at *JXB* online). These results indicated that suboptimal ploidy can reduce chilling tolerance in Mxg even when advantageous genes and gene combinations are present. In previous studies, hexaploids of Mxg had slightly greater stem diameter, slower growth rates (Yu *et al.*, 2009), and fewer shoots (Głowacka *et al.*, 2010), compared with triploid Mxg. Thus, ploidy can affect a range of traits, including chilling tolerance.

### What variation in chilling tolerance can be found in Mxg’s parental species?

Based on the present sampling, Msa was typically a better source of chilling tolerance than Msi (Figs 1, 2; Supplementary Fig. S1 at *JXB* online), though Msi from particularly cold environments in the northernmost part of the species natural range, such as northern China, and southeast Russia were not available for this study. The results were consistent with the fact that the natural range of Msa extends further north in Asia than Msi, indicating that Msa is generally better adapted to colder temperate environments than Msi (Clifton-Brown *et al.*, 2008; Sacks *et al.*, 2013).

Though only a few other studies of low temperature leaf elongation or photosynthesis have been conducted on multiple genotypes of *Miscanthus*, they all support the conclusion that there is genetic variation within *Miscanthus* for chilling tolerance (Earnshaw *et al.*, 1990; Clifton-Brown and Jones, 1997; Farrell *et al.*, 2006; Purdy *et al.*, 2013). Farrell *et al.* (2006) compared four genotypes for leaf elongation of dormant rhizomes in the dark and found that Msi (2*x*) JESEL78 (a selection from the Jelitto population New Hybrid) was superior at all temperatures tested (7, 9, 11, 13, and 15 °C) to Mxg (3*x*), Msi (3*x*) ‘Goliath’ (GOFAL7), and Msa (4*x*) Sac-5 (=‘EMI-5’=MATEREC11). Purdy *et al.* (2013) studied leaf elongation at 12 °C for 4 d after transfer from 28 °C for four *Miscanthus* genotypes: Mxg (3*x*), Msi (3*x*) ‘Goliath’, Msa (4*x*) Sac-5, and Msi (2*x*) EMI-11. Purdy *et al.* (2013) observed no significant decrease in leaf elongation for Mxg, which may reflect the shorter duration of the chilling treatment than in the present study. All other genotypes in the Purdy *et al.* (2013) study had a significant decrease in leaf elongation relative to Mxg, which was similar to 32 (65%) genotypes tested in the present study. It is noteworthy that Msa (4*x*) EMI-5 was neither the best nor worst performing Msa under chilling conditions in the present study, but it was the only Msa included in the studies of Farrell *et al.* (2006) and Purdy *et al.* (2013). Clifton-Brown and Jones (1997) compared leaf elongation of Mxg with 21 accessions of Msi and four of Msa, and they observed that the best and worst genotypes under the lowest temperatures studied (5 °C for 11.5h) were Msi. It is unclear why the study of Clifton-Brown and Jones (1997) did not identify Mxg as a superior genotype under the lowest temperature treatment, in contrast to the study of Purdy *et al.* (2013) and the present study. Earnshaw *et al.* (1990) observed that *M. floridulus* originating from high altitude (3280 m) in Papua New Guinea had greater chilling tolerance than a low altitude (2600 m) accession, as evidenced by greater chlorophyll fluorescence after chilling of leaf samples at 0 °C, which indicated that natural selection for adaptation has resulted in genetic diversity of chilling tolerance in *Miscanthus*. An important contribution of the present study is that it was possible to evaluate more than twice as many Msa genotypes as all of the previous studies combined, which enabled the observation that both diploid and tetraploid genotypes of Msa typically have high, though variable tolerance to chilling.

In the present study, some Msa had chilling tolerance equivalent to Mxg (3*x*) ‘Illinois’. Tetraploid Msa ‘PF30153’, which was collected in Gifu Prefecture, did not differ significantly from Mxg (3*x*) ‘Illinois’ for chilling tolerance associated with leaf elongation and photosynthesis (Figs 1, 3, 4). Moreover, all Msa accessions studied, except the ornamental, chlorophyll mutant ‘Gotemba Gold’, did not show a significantly greater decline in the operating efficiency of photosystem II (Φ_PSII_) relative to Mxg (3*x*) ‘Illinois’ at the end of the 11 d chilling treatment and upon recovery to warm temperature (day 12) (Fig. 3; Supplementary Figs S2, S3). Photosystem II is considered the most sensitive part of the photosynthetic apparatus to light-induced chilling damage and often the first manifestation of chilling damage (Long *et al.*, 1994; Maxwell and Johnson, 2000). Thus, stability of the photosynthetic apparatus under cold stress is necessary but insufficient for high levels of photosynthesis *per se* at low temperature, yet the former trait may be of considerable value on its own. The ability to recover high rates of photosynthesis quickly upon return of warm temperatures may itself contribute to adaptation to fluctuating temperatures that are typical in temperate environments of the beginning and end of the growing season.

Given that Mxg (3*x*) ‘Illinois’ and most of the Msi included in the present study originated in southern Japan, yet chilling tolerance was highest for Mxg (3*x*) ‘Illinois’ and Msa, it is likely that Mxg’s chilling tolerance was obtained from its Msa parent. Furthermore, as the tetraploid parent to this triploid, Msa has double the influence of Msi on the genetic makeup of the Mxg hybrid. Given that chilling tolerance varies among and within *Miscanthus* species, careful selection of parents for developing new Mxg bioenergy cultivars will be needed to ensure optimal adaptation to temperate environments.

High productivity in a cool temperate climate requires a genotype to be able to develop photosynthetically competent leaves under the chilling conditions of early spring. Without this, a crop canopy will be unable to use the high insolation of April, May, and June fully in the northern hemisphere. This capacity is fundamental to explaining the higher productivity of Mxg (3*x*) ‘Illinois’ relative to *Z. mays* (Dohleman and Long, 2009; Long and Spence, 2013). Based on a productivity model, Farrell *et al.* (2006) predicted that a *Miscanthus* accession with greater cold and chilling tolerance than Mxg could extend the growing season an average of 30 d, which would result in up to 25% higher yield. Thus, understanding how to select for improved rates of photosynthesis under low temperatures is key to improving productivity and sustainability of crops grown in temperate environments, such as *Miscanthus*. For breeding Mxg, choice of both the Msa and Msi parents will be important for achieving gains in chilling tolerance and concomitant yield potential in temperate environments. Indeed, the four new triploid Mxg progeny [Msa (4*x*) ‘Bluemel Giganteus’*×*Msi (2*x*) var. *condensatus* ‘Cabaret’] that were tested in the leaf elongation experiment had inferior chilling tolerance relative to their Msa parent, because their Msi parent was poorly adapted to cold temperatures.

### What are the physiological mechanisms of chilling susceptibility and chilling tolerance of *Miscanthus* accessions?

All 16 accessions studied (13 *Miscanthus*, two sugarcane, and one maize) showed decreases in *A*_sat_, and Φ_PSII_, and increases in *c*_i_/*c*_a_, after transfer from 25 °C/20 °C to 10 °C/ 5 °C (Fig. 3; Supplementary Fig. S2 at *JXB* online). Stomatal conductance for most of the genotypes (81% of accessions) increased on day 0 and then decreased during the following 11 d of chilling treatment. Since in the intercellular compartment the concentration of CO_2_ increased as a consequence of a low rate of CO_2_ fixation, the stomata reacted by reducing their aperture. Thus, differences among accessions in relative decreases in *A*_sat_ cannot be explained by deficiency in CO_2_ or by loss of stomatal function. Comparing the *A*_sat_ reading with values of Φ_PSII_ showed that light-induced chilling damage of photosystem II is the primary reason for low CO_2_ assimilation in chilling-susceptible accessions. The most chilling-tolerant accessions retained relatively high Φ_PSII_ and *A*_sat_ during 11 d of chilling treatment and, upon return to warm temperatures (on day 12 of the experiment), they also recovered their gas exchange and chlorophyll fluorescence to pre-chilling treatment levels. These results are consistent with those of Farage *et al.* (2006), which indicated that the main mechanism behind avoiding the photodamage under chilling temperatures is the capacity for non-photochemical quenching of absorbed light energy. In contrast to the chilling-tolerant accessions, the chilling-susceptible accessions in the present study showed very low levels of recovery of measured parameters upon return to warm temperatures at the end of the experiment. This indicates a damaged photosynthetic system in susceptible accessions, and suggests the absence of alternative pathways for dissipating absorbed excitation energy under chilling conditions, leading to irreversible photo-oxidation of photosystem II (Allen and Ort, 2001).

## Conclusion

Based on the sampling carried out here, Msa is a better source of chilling tolerance than Msi; thus an even more extensive survey of tetraploid Msa collected from diverse environments would be advantageous for identifying superior Msa parents and breeding new chilling-tolerant Mxg cultivars. There is also a need to identify Msi parents with superior chilling tolerance, though this may be more challenging than for Msa. To the extent that lessons from breeding *Miscanthus* for increased photosynthetic capacity at low temperature can be applied to other crops, especially other *Saccharinae*, great gains in agricultural productivity and sustainability may be obtained in temperate environments.

## Supplementary data

Supplementary data are available at *JXB* online.

Figure S1. Relationship between leaf elongation at warm and chilling temperatures for 51 *Miscanthus* accessions, two control sugarcane, and two maize lines.

Figure S2. Leaf CO_2_ uptake rate, quantum yield of photosystem II, stomatal conductance to water vapour, and ratio of intercellular to atmospheric CO_2_ concentration for warm conditions prior to chilling treatment, after transfer of plants from warm to chilling (day 0), on the 11th day of chilling treatment, and 1 d after transfer of plants back to warm conditions. Numbers are expressed as a percentage of rates observed in warm conditions before the chilling treatment.

Figure S3. Changes in quantum yield of photosystem II and intercellular to atmospheric CO_2_ concentration following transfer of plants from warm to chilling conditions.

Supplementary Data

## References

[CIT0001] Allen DJ Ort DR 2001 Impacts of chilling temperatures on photosynthesis in warm-climate plants. Trends in Plant Science 6, 36–42. 11164376 10.1016/s1360-1385(00)01808-2

[CIT0002] Arundale RA Dohleman FG Heaton EA McGrath JM Voigt TB Long SP 2014 Yields of *Miscanthus × giganteus* and *Panicum virgatum* decline with stand age in the Midwestern USA. Global Change Biology Bioenergy 6, 1–13.

[CIT0003] Beale CV Bint DA Long SP 1996 Leaf photosynthesis in the C_4_-grass *Miscanthus×giganteus*, growing in the cool temperate climate of southern England. Journal of Experimental Botany 47, 267–273.

[CIT0004] Beale CV Long SP 1995 Can perennial C_4_ grasses attain high efficiencies of radiant energy conversion in cool climates? Plant, Cell and Environment 18, 641–650.

[CIT0005] Bernacchi CJ Pimentel C Long SP 2003 *In vivo* temperature response functions of parameters required to model RuBP-limited photosynthesis. Plant, Cell and Environment 26, 1419–1430.

[CIT0006] Chae WB Hong SJ Gifford JM Rayburn AL Sacks EJ Juvik JA 2014 Plant morphology, genome size and SSR markers differentiate five distinct taxonomic groups among accessions in the genus *Miscanthus* . Global Change Biology Bioenergy (in press).

[CIT0007] Chae WB Hong SJ Gifford JM Rayburn AL Widholm JM Juvik JA 2013 Synthetic polyploid production of *Miscanthus sacchariflorus, Miscanthus sinensis*, and *Miscanthus×giganteus* . Global Change Biology Bioenergy 5, 338–350.

[CIT0008] Chen YH Chen C Lo CC 1993 Studies on anatomy and morphology in *Saccharum–Miscanthus* nobilized hybrids.1. Transmission of tillering, ratooning, adaptation and disease resistance from *Miscanthus* spp. Journal of the Agricultural Association of China 31–45.

[CIT0009] Clifton-Brown JC Chiang Y-C Hodkinson TR 2008 *Miscanthus*: genetic resources and breeding potential to enhance bioenergy production. In: Vermerris W, ed. Genetic improvement of bioenergy crops. New York: Springer, 273–294.

[CIT0010] Clifton-Brown JC Jones MB 1997 The thermal response of leaf extension rate in genotypes of the C_4_-grass *Miscanthus*: an important factor in determining the potential productivity of different genotypes. Journal of Experimental Botany 48, 1573–1581.

[CIT0011] Clifton-Brown JC Stampfl PF Jones MB 2004 *Miscanthus* biomass production for energy in Europe and its potential contribution to decreasing fossil fuel carbon emissions Global Change Biology 10, 509–518.

[CIT0012] Dohleman FG Long SP 2009 More productive than maize in the Midwest: how does Miscanthus do it? Plant Physiology 150, 2104–2115. 19535474 10.1104/pp.109.139162 PMC2719137

[CIT0013] Dwiyanti MS Rudolph A Swaminathan K 2013 Genetic analysis of putative triploid *Miscanthus* hybrids and tetraploid *M. sacchariflorus* collected from sympatric populations of Kushima, Japan. BioEnergy Research 6, 486–493.

[CIT0014] Earnshaw MJ Carver KA Gunn TC Kerenga K Harvey V Griffiths H Broadmeadow MSJ 1990 Photosynthetic pathway, chilling tolerance and cell sap osmotic potential values of grasses along an altitudinal gradient in Papua New Guinea. Oecologia 84, 280–288. 10.1007/BF00318285 28312766

[CIT0015] Farage PK Blowers D Long SP Baker NR 2006 Low growth temperatures modify the efficiency of light use by photosystem II for CO_2_ chilling-tolerant C_4_ assimilation in leaves of two species, *Cyperus longus* L. and *Miscanthus* × *giganteus* . Plant, Cell and Environment 29, 720–728. 10.1111/j.1365-3040.2005.01460.x 17080621

[CIT0016] Farrell AD Clifton-Brown JC Lewandowski I Jones MB 2006 Genotypic variation in cold tolerance influences the yield of Miscanthus. Annals of Applied Biology 149, 337–345.

[CIT0017] Głowacka K Clark LV Adhikari S 2014 Genetic variation in Miscanthus ×giganteus and the importance of estimating genetic distance thresholds for differentiating clones. Global Change Biology Bioenergy (in press).

[CIT0018] Głowacka K Jeżowski S Kaczmarek Z 2010 *In vitro* induction of polyploidy by colchicine treatment of shoots and preliminary characterisation of induced polyploids in two *Miscanthus* species. Industrial Crops and Products 32, 88–96.

[CIT0019] Greef JM Deuter M 1993 Syntaxonomy of *Miscanthus* ×*giganteus* Greef et Deu. Angewandte Botanik 67, 87–90.

[CIT0020] Heaton E Dohleman FG Long SP 2008 Meeting US biofuel goals with less land: the potential of *Miscanthus* . Global Change Biology 14, 1–15.

[CIT0021] Hodkinson TR Chase MW Takahashi C Leitch IJ Bennett MD Renvoize SA 2002 The use of DNA sequencing (ITS and t*rnL-F*) AFLP and fluorescent *in situ* hybridization to study allopolyploid *Miscanthus* (Poaceae). American Journal of Botany 89, 279–286. 21669737 10.3732/ajb.89.2.279

[CIT0022] Hodkinson T Renvoize S 2001 Nomenclature of *Miscanthus* ×*giganteus* (*Poaceae*). Kew Bulletin 56, 759–760.

[CIT0023] Jeżowski S Głowacka K Kaczmarek Z 2011 Variation on biomass yield and morphological traits of energy grasses from the genus *Miscanthus* during the first years of crop establishment. Biomass and Bioenergy 35, 814–821.

[CIT0024] Lewandowski I Clifton-Brown JC Scurlock JMO Huisman W 2000 *Miscanthus*: European experience with a novel energy crop. Biomass and Bioenergy 19, 209–227.

[CIT0025] Linde-Laursen IB 1993 Cytogenetic analysis of *Miscanthus* ‘Giganteus’, an interspecific hybrid. Hereditas 119, 297–300.

[CIT0026] Long SP 1983 C_4_ photosynthesis at low temperatures. Plant, Cell and Environment 6, 345–363.

[CIT0027] Long SP Humphries S Falkowski PG 1994 Photoinhibition of photosynthesis in nature. Annual Review of Plant Physiology and Plant Molecular Biology 45, 633–662.

[CIT0028] Long SP Spence AK 2013 Toward cool C_4_ crops. Annual Review of Plant Biology 64, 701–722. 10.1146/annurev-arplant-050312-120033 23473604

[CIT0029] Maxwell K Johnson GN 2000 Chlorophyll fluorescence—a practical guide. Journal of Experimental Botany 51, 659–668. 10938857 10.1093/jxb/51.345.659

[CIT0030] Naidu SL Long SP 2004 Potential mechanisms of low-temperature tolerance of C_4_ photosynthesis in *Miscanthus*×*gigantues*: an *in vivo* analysis. Planta 220, 145–155. 15258759 10.1007/s00425-004-1322-6

[CIT0031] Naidu SL Moose SP AL-Shoaibi AK Raines CA Long SP 2003 Cold tolerance of C_4_ photosynthesis in *Miscanthus*×*giganteus*: adaptation in amounts and sequence of C_4_ photosynthetic enzymes. Plant Physiology 132, 1688–1697. 12857847 10.1104/pp.103.021790 PMC167105

[CIT0032] Nishiwaki A Mizuguti A Kuwabara S 2011 Discovery of natural *Miscanthus* (Poaceae) triploid plants in sympatric populations of *Miscanthus sacchariflorus* and *Miscanthus sinensis* in Southern Japan. American Journal of Botany 98, 154–159. 21613094 10.3732/ajb.1000258

[CIT0033] Purdy SJ Maddison AL Jones LE Webster RJ Andralojc J Donnison I Clifton-Brown J 2013 Characterization of chilling-shock responses in four genotypes of *Miscanthus* reveals the superior tolerance of *M*.×*giganteus* compared with *M. sinensis* and *M. sacchariflorus* . Annals of Botany 111, 999–1013. 23519835 10.1093/aob/mct059 PMC3631343

[CIT0034] Sacks EJ Juvik JA Lin Q Stewart JR Yamada T 2013 The gene pool of *Miscanthus* species and its improvement. In: Paterson AH, ed. Genomics of the Saccharinae . New York: Springer, 73–101.

[CIT0035] Scurlock JMO 1999 *Miscanthus*: a review of European experience with a novel energy crop. In: ORNL Technical Memorandum TM-13732 . Oak Ridge, TN: Oak Ridge National Laboratory, 18.

[CIT0036] Somerville C Youngs H Taylor C Davis SC Long SP 2010 Feedstocks for lignocellulosic biofuels. Science 329, 790–792. 20705851 10.1126/science.1189268

[CIT0037] Wang D Naidu SL Portis AR Jr Moose SP Long SP 2008a Can the cold tolerance of C_4_ photosynthesis in *Miscanthus*×*giganteus* relative to *Zea mays* be explained by differences in activities and thermal properties of Rubisco? Journal of Experimental Botany 59, 1779–1787. 18503044 10.1093/jxb/ern074

[CIT0038] Wang D Portis AR Jr Moose SP Long SP 2008b Cool C_4_ photosynthesis: pyruvate Pi dikinase expression and activity corresponds to the exceptional cold tolerance of carbon assimilation in *Miscanthus*×*giganteus* . Plant Physiology 148, 557–567. 18539777 10.1104/pp.108.120709 PMC2528129

[CIT0039] Yu CY Kim HS Burn AL Widholm JM Juvik John A. 2009 Chromosome doubling of the bioenergy crop, *Miscanthus*×*giganteus* . Global Change Biology Bioenergy 1 404–412.

