# Supplementary material for: Variation in chilling tolerance for photosynthesis and leaf extension growth among genotypes related to the C4 grass Miscanthus ×giganteus
Source: J Exp Bot. 2014 Jul 19;65(18):5267–78. doi: 10.1093/jxb/eru287 (PMC4157708; doi:10.1093/jxb/eru287)
Supplement: Supplementary Data [file supp_65_18_5267__index.html]

Variation in chilling tolerance for photosynthesis and leaf extension growth among genotypes related to the C4 grass Miscanthus ×giganteus — Variation in chilling tolerance for photosynthesis and leaf extension growth among genotypes related to the C4 grass Miscanthus ×giganteus — Supplementary Data 

# Variation in chilling tolerance for photosynthesis and leaf extension growth among genotypes related to the C4 grass *Miscanthus ×giganteus*

## Supplementary Data

Data files

**Files in this Data Supplement:**

- Supplementary Data - Supplementary Data
