# Supplementary material for: Variation in chilling tolerance for photosynthesis and leaf extension growth among genotypes related to the C4 grass Miscanthus ×giganteus
Source: J Exp Bot. 2014 Jul 19;65(18):5267–78. doi: 10.1093/jxb/eru287 (PMC4157708; doi:10.1093/jxb/eru287)
Supplement: Supplementary Data [file supp_eru287_jexbot119511_file001.pdf]

Variation in chilling tolerance for photosynthesis and leaf extension growth among genotypes related to the C_4 grass *Miscanthus ×giganteus*.

Katarzyna Głowacka, Shivani Adhikari, Junhua Peng, Justin Gifford, John A. Juvik, Stephen P. Long, Erik J. Sacks

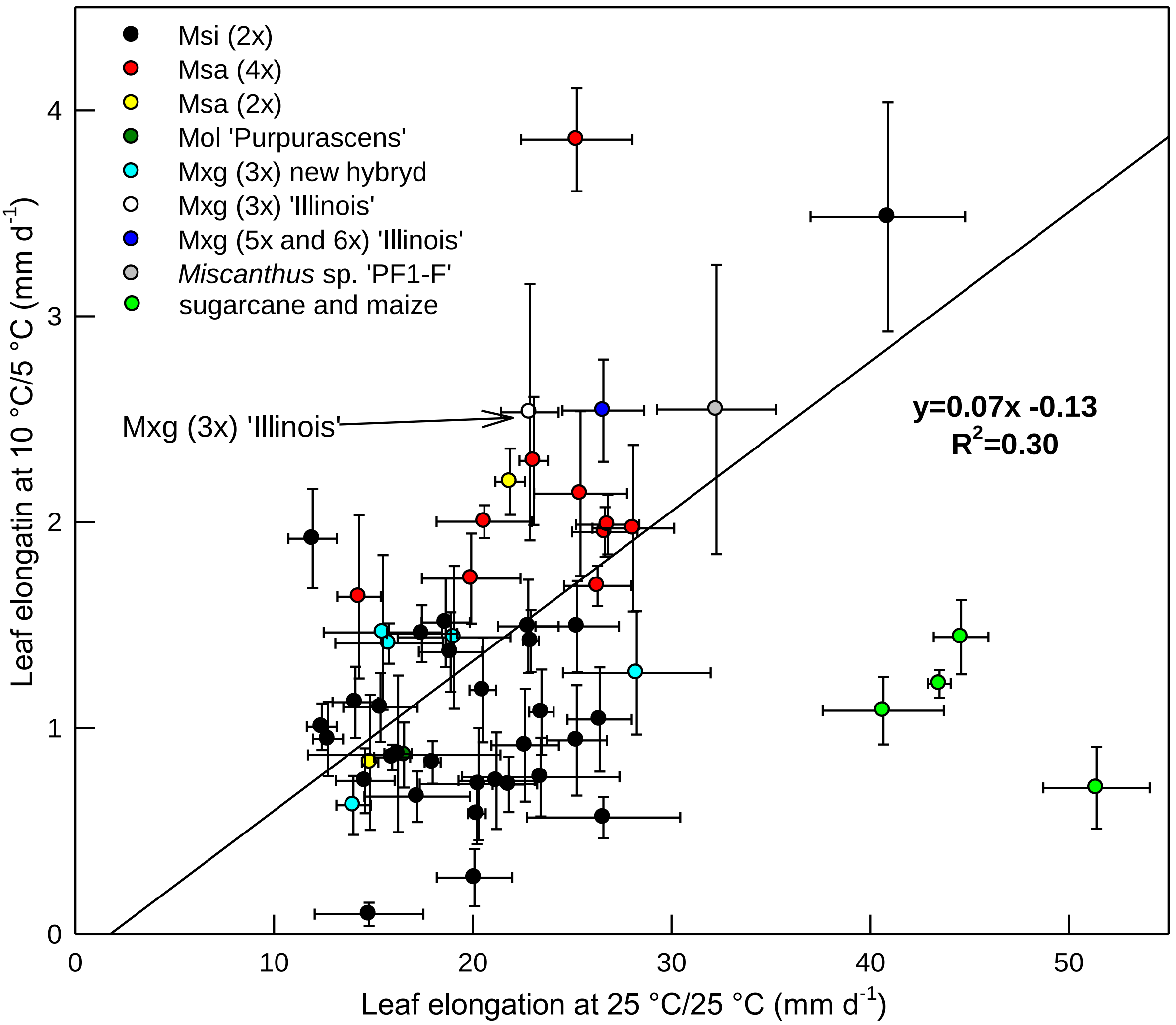

Fig. S1. Relationship between leaf elongation in warm and chilling temperature for 51 *Miscanthus* accessions, 2 control sugarcane and 2 maize lines. Plants were grown at 25 °C/25 °C (warm) or 10 °C/5 °C (chilling) day/night, and 12-h-day/12-h-night cycle under 1000 µmol photons m^{-2} s^{-1}. Measurements were taken during the day. In chilling, developing leaves were measured during 14 days every other day, while for warm conditions data was collected during 7 days every day. Line presents linear regression for *Miscanthus* accessions. Data are mean ± SE (n=3). Mol= *M. oligostachyus*; Msa = *M. sacchariflorus*; Msi = *M. sinensis*; Mxg = *M. ×giganteus*.

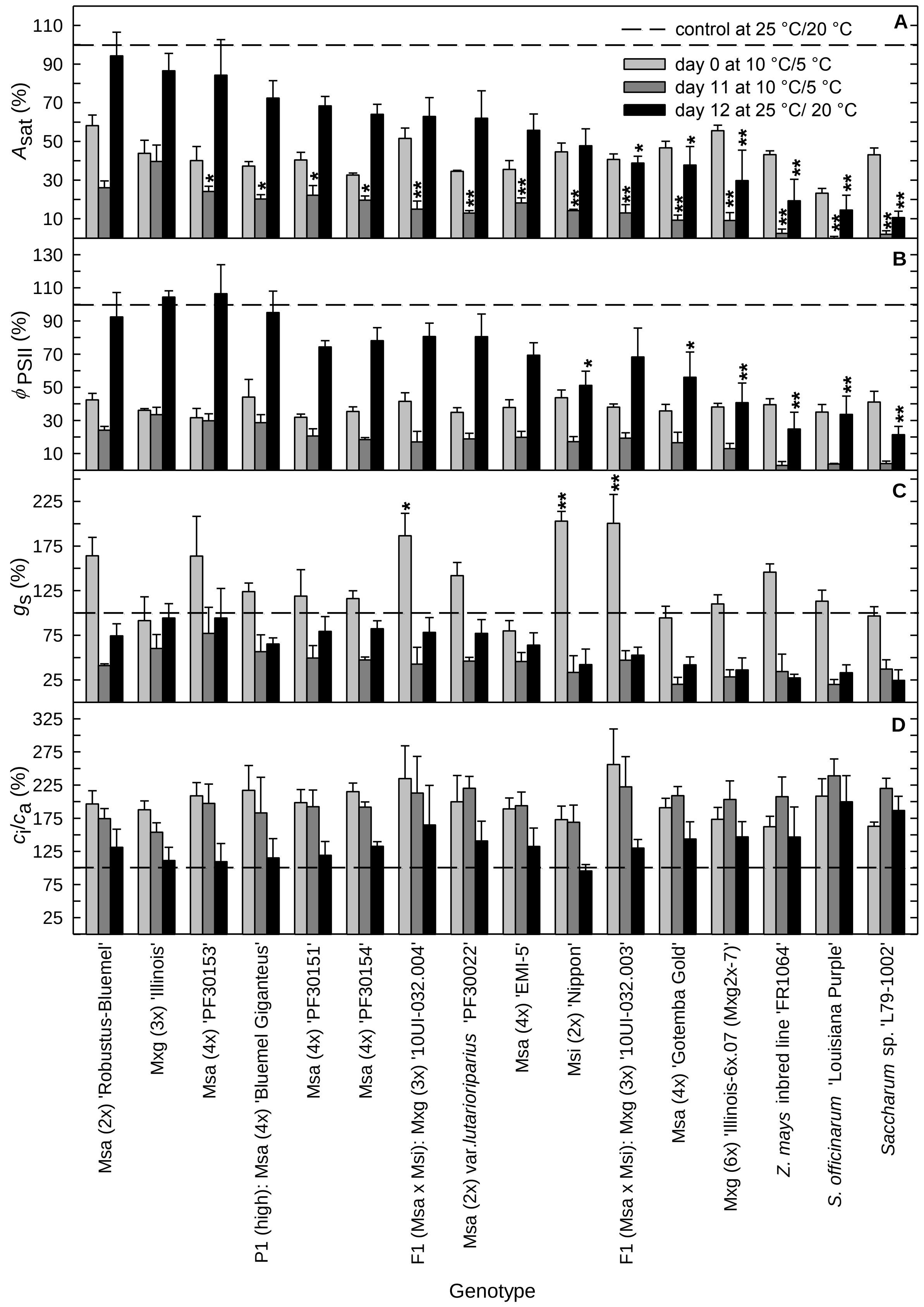

Fig. S2. (A) Leaf CO_2 uptake rate (A_{sat}), (B) quantum yield of photosystem II (Φ_{PSII}), (C) stomatal conductance to water vapor (g_s) and (D) ratio of intercellular to atmospheric CO_2

concentration (c_i/c_a) for warm conditions prior to chilling treatment, after transfer of plants from warm to chilling (day 0), in 11th day of chilling treatment and one day after transfer plants back to warm (12th day of experiment - recovery). Numbers are expressed as a percentage of rates observed in warm conditions before the chilling treatment. Plants were grown at 10 °C/5 °C (chilling) or 25 °C/20 °C (warm) day/night, and 14-h-day/10-h-night cycle under 1000 μmol photons m^{-2} s^{-1}. In all panels accessions are ordered according to A_{sat} on day 12th of experiment (from highest to lowest; panel A, third bar (black fill) for each genotype). Measurements were taken during day time. For each treatment stage, asterisks indicate significant differences in comparison to *M.* ×*giganteus* (3x) 'Illinois' based on Dunnett's test (*≤0.05; **≤0.01). Subsequent-time-point values for Mxg (3x) 'Illinois' were: (A) 43.86, 39.64 and 86.54 (%); (B) 36.09, 33.49 and 104.45 (%); (C) 91.36, 60.19 and 94.40 (%); (D) 187.70, 153.96 and 111.34 (%). Data are mean + SE (n=4). F1 = the first generation of Msa × Msi hybrids; Msa = *M. sacchariflorus*; Msi= *M. sinensis;* Mxg = *M.* ×*giganteus*; P1 (high) = parent 1 of interspecific Msa × Msi hybrids (Msa with high chilling tolerance).

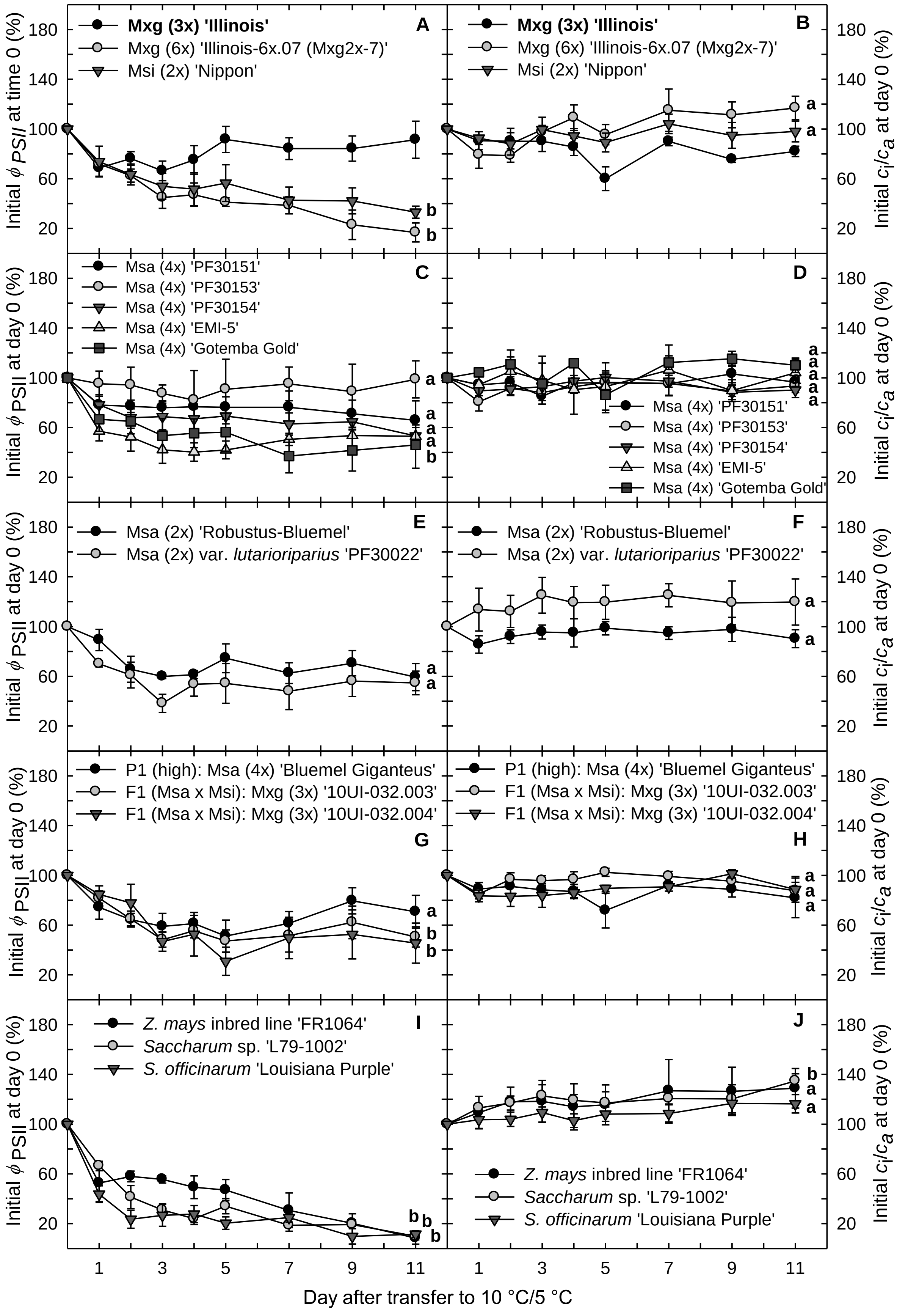

Fig. S3. Changes in: (A; C; E; G; I) quantum yield of photosystem II (Φ_{PSII}) and (B; D; F; H; J) intercellular to atmospheric CO_2 concentration (c_i/c_a) following transfer of plants from

warm to chilling conditions. Values are expressed as a percentage of initial rates at time 0. (A–B) accessions at different ploidy levels; (C–D) tetraploid *M. sacchariflorus* (Msa); (E–F) diploid Msa; (G–H) interspecific hybrids (F1) and their Msa parent (P1; high); (I–J) negative controls. Plants were grown at 25 °C/20 °C (warm) or 10 °C/5 °C (chilling) day/night, and 14-h-day/10-h-night cycle under 1000 μmol photons m^{-2} s^{-1}. Measurements were taken during day time. Data are mean ± SE (n=4). Low case letters indicate: (“a”) not significant differences or (“b”) significant differences in comparison to *M.* ×*giganteus* (3x) ‘Illinois’ (bold) on day 11th after transfer to 10 °C/5 °C on the based on Dunnett’s test ($p \leq 0.05$). Values for Mxg (3x) ‘Illinois’ on the day 11th of chilling treatment were: (A) 91.40%; (B) 116.88%. F1 = the first generation of Msa × Msi hybrids; Msa = *M. sacchariflorus*; Msi = *M. sinensis*; Mxg = *M.* ×*giganteus*; P1 (high) = parent 1 of interspecific Msa × Msi hybrids (Msa with high chilling tolerance).
